# Temporal orientation and the teaching life cycle: pathways linking teachers’ growth, recognition, and performance across two studies

**DOI:** 10.3389/fpsyg.2025.1720811

**Published:** 2026-01-09

**Authors:** LingYan Meng, Bárbara Briscioli

**Affiliations:** School of Philosophy and Literature, University of Buenos Aires, Buenos Aires, Argentina

**Keywords:** achievement recognition, China, professional growth, secondary education, teaching performance, temporal orientation

## Abstract

This research examines how professional growth, achievement recognition, and temporal orientation interact to shape teaching performance among Chinese secondary school teachers. Two complementary studies were conducted: Study 1, a cross-sectional survey, and Study 2, an experiment manipulating temporal orientation. Study 1, involving 234 teachers, showed that professional growth was positively associated with teaching performance and that a small but statistically significant indirect effect via achievement recognition emerged, despite uncertainty in the strength of the path from professional growth to recognition. Study 2, with 253 participants, showed that experimentally inducing a long-term (vs. short-term or control) time orientation increased mean levels of recognition, professional growth, and teaching performance, but did not support the hypothesized moderation of the mediation mechanism. Overall, findings indicate that professional growth is a consistent predictor of teaching performance, recognition serves as a modest psychosocial pathway, and temporal orientation operates primarily as a contextual enhancer of mean levels rather than as a structural moderator of the mediation process. The study contributes to theoretical and practical understanding of how teacher development, recognition practices, and future-oriented thinking can jointly sustain motivation and effectiveness within the Chinese secondary education system.

## Introduction

Most of us can recall the figure of a secondary school teacher who left a decisive mark during adolescence, a period often described as turbulent and formative. At a stage when young people are negotiating the challenges of identity, relationships, and future aspirations, the presence of a teacher who fosters learning and personal growth can shape not only academic achievement but also long-term development (Wu et al., 2025). Yet while teachers exert such influence on their students, it is equally important to recognize that their own professional trajectory is closely intertwined with the quality of their teaching performance (Yao et al., 2024).

In China, secondary school teachers face increasing demands to meet performance standards while navigating systemic reforms, accountability pressures, and rapid social change (Zou and Song, 2025). Against this backdrop, questions of how teachers grow professionally, how their achievements are recognized, and how these processes influence their performance have become central for both researchers and policymakers. Understanding these dynamics is especially relevant in contexts where the teaching profession is both highly valued culturally and challenged by structural inequities, such as the urban–rural divide (Cheng et al., 2025).

Professional Growth is commonly regarded as the ongoing process by which teachers enhance their skills, knowledge, and professional identity through training, practice, and reflection. In the Chinese context, professional development has been deeply institutionalized through structures such as teaching–research groups and district-level support systems, which foster collegial learning and peer feedback (Wang et al., 2024). Yet, while opportunities for growth are widely available, the extent to which these experiences translate into improved classroom performance depends on more than the acquisition of skills alone. Teachers must also feel that their efforts are seen and valued (Kong et al., 2024).

Achievement Recognition, understood as the acknowledgment of teachers’ contributions and accomplishments, represents one of the key psychosocial mechanisms that connect growth to performance. Recognition can take many forms, ranging from formal honors and promotions to informal appreciation from colleagues and students. When teachers perceive that their achievements are recognized, they are more likely to internalize the benefits of professional growth, reinforcing their motivation, sense of belonging, and commitment to the profession (Meng and Briscioli, 2024). From a psychological standpoint, these mechanisms resonate with Self-Determination Theory (Ryan and Deci, 2017), which posits that the fulfillment of basic psychological needs—autonomy, competence, and relatedness—enhances intrinsic motivation and performance. Recognition contributes directly to teachers’ perceived competence and social relatedness, thereby transforming professional development experiences into enduring motivational gains. At the same time, Social Cognitive Theory (Bandura, 1993) suggests that professional growth strengthens self-efficacy through mastery experiences and social modeling. When recognition follows professional improvement, it provides social reinforcement that validates teachers’ self-efficacy beliefs, further encouraging persistence and performance. Hence, growth and recognition are not isolated organizational features but intertwined motivational processes that sustain engagement and behavioral effectiveness.

Teaching performance, operationalized in this study as task performance, represents the extent to which teachers effectively carry out the core duties of their role. Task performance refers to behaviors that are formally recognized as part of the job, such as lesson preparation, classroom delivery, and evaluation of students (Motowidlo et al., 2014). Prior research has consistently shown that teachers who invest in their professional growth tend to perform better in these core tasks, although the strength of this relationship varies depending on contextual and personal factors (Zhang et al., 2023). Achievement recognition appears to be one of the most salient of these factors, serving as a bridge that allows growth to be translated into effective performance (Renger et al., 2020).

In addition to growth and recognition, teachers’ temporal orientation adds a further layer to this dynamic. In this article, temporal orientation is used as an umbrella term to describe how individuals mentally organize their experience of time in work and career. Within this broader category, we focus on future-oriented temporal orientation, commonly operationalized as future time perspective (FTP). A stronger future-oriented perspective encourages sustained investment in professional growth and greater openness to recognition processes, whereas a short-term or present-focused perspective may narrow attention to immediate demands and reduce the motivational impact of recognition (Zacher and Frese, 2009; Zacher et al., 2010; Rudolph et al., 2018). In Study 1, temporal orientation is measured using a future time perspective scale, whereas Study 2 employs a broader time-perspective measure while experimentally priming a long-term, future-oriented focus. For clarity, we use temporal orientation when referring to the general construct and future time perspective when discussing the specific operationalization used in Study 1.

Bringing these elements together, the present research focuses on a core pathway in which professional growth predicts teaching performance, and achievement recognition functions as a key psychosocial mechanism linking the two. When teachers invest in their own development, they expand their instructional knowledge and skills; however, these gains are most likely to translate into higher performance when teachers feel that their efforts are noticed and valued. Recognition thus forms the psychological bridge through which growth experiences are internalized and enacted in daily teaching practice. Importantly, achievement recognition is examined as a complementary and potentially weak psychosocial pathway, rather than as a central or exhaustive mechanism explaining the association between professional growth and teaching performance.

At the same time, temporal orientation is conceptualized as a boundary condition that may shape how strongly this growth–recognition–performance mechanism operates and how elevated these resources are at the mean level. A long-term, future-oriented temporal orientation may strengthen teachers’ willingness to pursue development, remain receptive to recognition, and maintain effort toward performance goals, whereas a more short-term focus may limit these processes. Accordingly, Study 1 examines whether future time perspective moderates the indirect association from professional growth to teaching performance via achievement recognition, and Study 2 tests whether experimentally inducing a long-term temporal orientation enhances average levels of growth, recognition, and performance relative to short-term and control conditions.

The present research therefore seeks to advance understanding of these interrelated processes by examining how professional growth predicts teaching performance, how achievement recognition mediates this association, and how temporal orientation can either moderate the strength of these pathways or enhance overall levels of growth, recognition, and performance. By conducting two complementary studies—a cross-sectional analysis and an experimental intervention—we integrate correlational and experimental evidence within a single framework. This study extends existing models of teacher motivation by positioning recognition as a psychosocial mediator between growth and performance and by testing the conditions under which temporal orientation shapes these processes within Chinese secondary schools.

## Literature review and hypotheses development

### Theoretical framework: a psychosocial perspective

The present research is grounded in major psychological theories that explain how motivation and recognition shape professional behavior. From the lens of Self-Determination Theory (Ryan and Deci, 2017), teachers’ motivation to grow and perform effectively depends on the fulfillment of basic psychological needs for competence, autonomy, and relatedness. Professional growth enhances perceived competence, while achievement recognition strengthens relatedness and reinforces intrinsic motivation. In parallel, Social Cognitive Theory (Bandura, 1993) emphasizes the role of self-efficacy and reciprocal determinism in explaining performance. When teachers perceive that their growth is acknowledged by others, this recognition validates their efficacy beliefs and sustains behavioral engagement. Together, these perspectives frame professional growth, recognition, and temporal orientation as interdependent psychosocial processes that sustain teachers’ motivation and performance.

### Professional growth and teaching performance

Professional growth refers to the continuous development of teachers’ knowledge, skills, and professional identity through training, practice, and reflection (Avalos, 2011). In China, teacher development has been deeply institutionalized through school-based teaching–research groups and district-level support offices, which create opportunities for collective lesson planning, peer observation, and collegial reflection (Li and Xue, 2023). Research consistently shows that professional growth is positively associated with teachers’ performance in fulfilling core job tasks, often described as task performance (Zhang et al., 2025). In this study, teaching performance is conceptualized as a self-perceived construct reflecting teachers’ subjective evaluation of how effectively they carry out core instructional and professional tasks. Although task performance is traditionally defined in behavioral terms—covering actions such as lesson preparation, classroom management, and student assessment—our operationalization relies on teachers’ self-reports rather than external observations. This approach captures teachers’ perceptions of their own task-related behaviors and has been widely used in educational and organizational psychology as a valid proxy for actual performance when direct behavioral indicators are not available (Koopmans et al., 2014; Wu et al., 2019). Task performance captures the extent to which teachers complete duties such as lesson preparation, classroom delivery, and student evaluation, which are central to their professional role (Li et al., 2018).

At the same time, the translation of growth into measurable task performance is not automatic. Contextual and psychological factors, such as the availability of supportive leadership or the perception of being valued, play a significant role in determining whether growth initiatives produce real improvements in teachers’ daily practice (Bautista et al., 2022). Taken together, this body of evidence suggests that professional growth can be a powerful driver of task-based teaching performance, but its effectiveness depends on additional conditions that shape how growth is enacted in the school context.

### Achievement recognition as a mediator

Teachers’ professional development is unlikely to translate fully into higher performance if their achievements are not acknowledged. Achievement recognition encompasses both formal mechanisms, such as promotions and awards, and informal expressions of appreciation from students, colleagues, and leaders (Honneth, 1995). Recognition practices foster motivation, belonging, and commitment, reinforcing teachers’ sense of professional honor. In the Chinese context, recognition has been shown to influence career persistence and mobility: teachers who feel their contributions are valued are more likely to remain in their positions, while those who lack recognition are at higher risk of attrition (Chen et al., 2025; (Peng and Nair, 2022)).

Research on transformational leadership in Chinese schools also highlights recognition as central to translating professional growth into effective teaching (Yu and Jang, 2024). From a psychological perspective, recognition satisfies fundamental needs for esteem and belonging (Bandura, 1993; Ryan and Deci, 2017), which in turn increase the likelihood that teachers apply newly acquired skills in their classrooms. Recent work similarly shows that recognition strengthens the connection between professional development and performance outcomes (Meng and Briscioli, 2024). Overall, this literature indicates that achievement recognition may serve as a crucial mechanism through which professional growth leads to improved performance.

### Time perspective as a boundary condition

As noted earlier, temporal orientation captures the way individuals construe their professional future. A future-oriented temporal orientation encourages sustained investment in professional growth and openness to developmental feedback and recognition, whereas a present-focused outlook may narrow attention to immediate tasks and reduce the motivational impact of recognition (Zimbardo and Boyd, 2015; Kooij et al., 2018). Prior research links future orientation to work motivation, adaptability, and career planning (Guo et al., 2022; Hanna et al., 2023; Mohsin et al., 2024), suggesting that teachers who perceive a broader future horizon may be especially likely to translate growth opportunities and recognition experiences into higher performance. From this perspective, temporal orientation can be understood as a boundary condition that may strengthen—or weaken—the indirect effect of professional growth on teaching performance through achievement recognition.

In educational contexts, future orientation has been linked to teachers’ commitment to professional development and their ability to cope with stress (Hanna et al., 2023; Rowe and Walinga, 2020). Studies in China show that adopting a longer temporal horizon can buffer the relationship between job strain and burnout, suggesting that envisioning a broader professional future helps teachers sustain motivation and resilience (Guo et al., 2022). However, little is known about whether temporal orientations alter the mechanisms connecting growth, recognition, and performance in secondary education. The available evidence suggests that time perspective—particularly a focus on the future—could shape how professional growth and recognition interact, though empirical results remain inconclusive.

### Experimental extension: manipulating temporal orientation

Most studies have conceptualized temporal orientation as a relatively stable individual difference. Yet research also indicates that orientations toward time can be influenced through interventions, such as exercises that emphasize long-term goals (Kooij et al., 2014). In the present research, we examined whether experimentally manipulating teachers’ temporal focus would influence their sense of achievement recognition and the translation of professional growth into teaching performance. Although the measure employed in Study 2 (the ZTPI-C) captures multiple dimensions of time perspective, the intervention specifically aimed to activate future-oriented thinking. Experimental designs thus provide a useful way to test whether shifting teachers’ temporal orientation toward the future can support professional development and performance.

## Study 1

**Figure 1:**
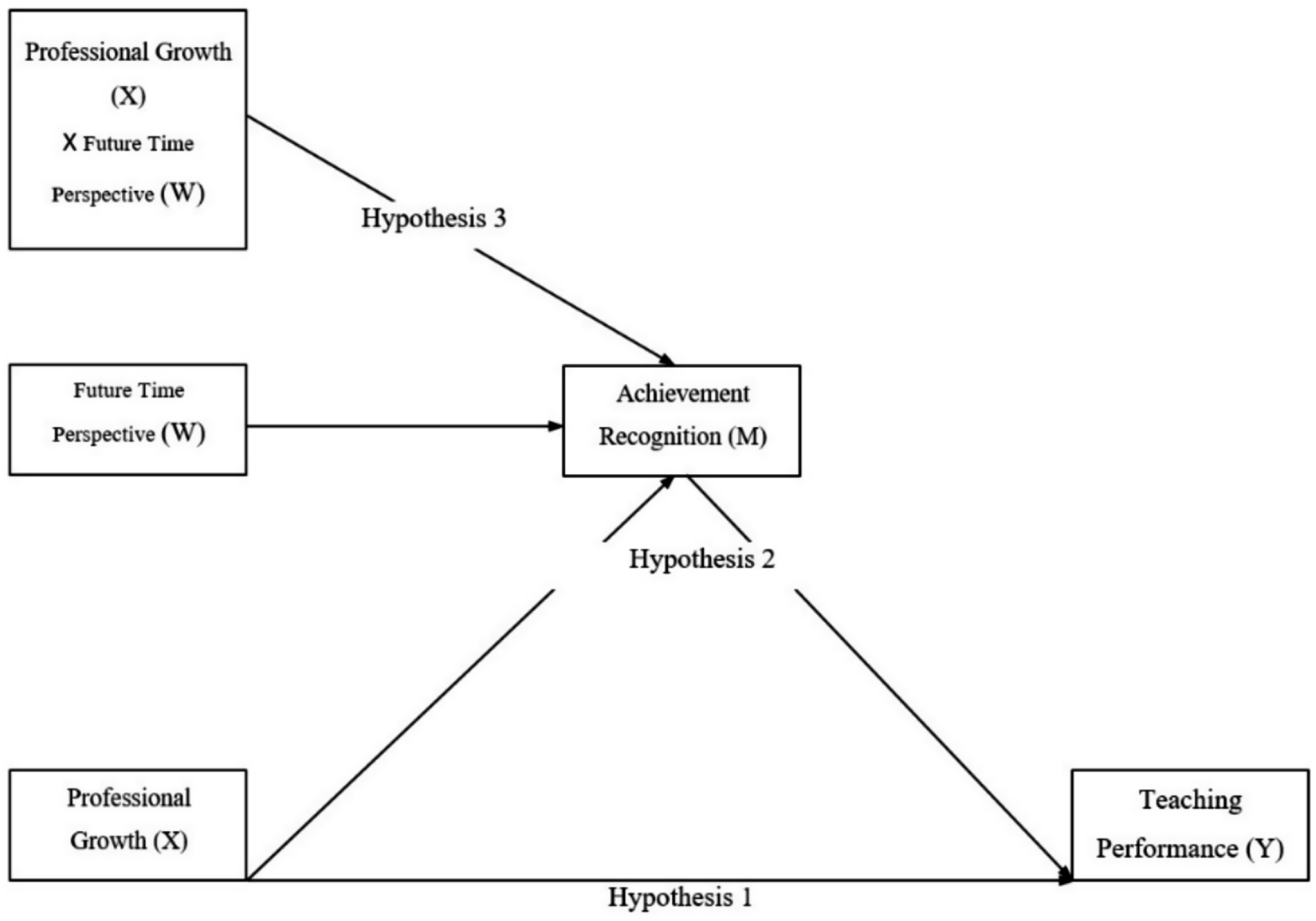
Conceptual model. Professional growth is expected to predict teaching performance directly and indirectly via achievement recognition, with the indirect path moderated by future time perspective.

Building on the arguments developed above, we first expected that teachers who perceive higher levels of professional growth would report higher task-based teaching performance (H1). We further anticipated that achievement recognition would mediate this association, such that professional growth would predict greater recognition, which in turn would predict better performance (H2). Finally, consistent with the view of temporal orientation as a boundary condition, we expected that future time perspective would moderate this indirect effect, with the pathway from professional growth through recognition to performance being stronger among teachers with higher future orientation (H3) (Figure 1).

### Method

#### Participants

Participants were 234 Chinese secondary school teachers (56.8% men; M_age = 34.92, SD = 8.31; range = 23–56). Regarding role, 5.6% were school managers, 36.3% heads of department, 27.9% tutors, and 22.9% teachers; the remaining 7.3% reported other or unspecified roles. In terms of teaching experience, 16.9% had less than 1 year, 8.7% had between one and 2 years, 29.7% had between 3 and 5 years, and 44.6% had more than 5 years. The dataset did not include additional contextual descriptors such as disciplinary specialization, regional location, or school-level indicators (e.g., urban/rural status or school tier).

#### Procedure

The design of the study adhered to the principles outlined in the Declaration of Helsinki and its further amendments (Fortaleza, Brazil) and complied with local legislation. The Institutional Review Board of the University of Buenos Aires provided Ethical approval prior to the study’s initiation, with protocol approval number [Approval Number: 2024-5668-PhiLit], granted on [June, 28th 2024]. Informed consent was obtained from all participants prior to their inclusion. The consent process included information about the study’s aim, its voluntary nature, the right to withdraw at any time without penalty, and the assurance of anonymity in responses. The study was disseminated through online platforms and mass emailing, specifically targeting secondary schools in China in order to reach potential participants. A total of 234 Chinese secondary school teachers aged 23–56 years were recruited using a non-probability purposive sampling technique. The choice of this sampling method was guided by the specific need to gather insights from teachers within a defined age range and educational sector, allowing for a focused exploration of the research questions. All data were collected through a secure online platform that did not record identifying information beyond participants’ voluntary demographic responses. No IP addresses or device identifiers were logged. Data were stored on encrypted, password-protected institutional servers accessible only to the research team. Because the authors are based in different countries, data handling procedures complied with the jurisdictional requirements of both institutions, including cross-border data-transfer regulations.

All questionnaires were administered in Chinese. Instruments originally published in English were translated and back-translated by two independent bilingual researchers following established guidelines (Klotz et al., 2023). This procedure ensured linguistic equivalence and cultural appropriateness across all measures. Several example items in both English and Chinese are provided in Appendix A to illustrate the translation decisions and ensure transparency.

#### Data availability statement

Anonymized data and analysis scripts for Study 1 are publicly available on the Open Science Framework (OSF) at the following link: https://osf.io/cnv5g/?view_only=772488d1fbb4475fb5ea9dbce3c1cd5b.

#### Instruments

##### Professional growth

In Study 1, professional growth was assessed using the Career Growth Scale for Nurses (Ni et al., 2023), adapted to the context of secondary school teachers. The instrument includes 17 items addressing different aspects of career development, such as improving professional abilities, advancing in one’s career, and making progress toward professional goals (e.g., “I gradually realize my role as a teacher and can better adapt to it,” “I carry out my career development plans conscientiously.”). Teachers responded on a 5-point Likert scale ranging from 1 (Strongly disagree) to 5 (Strongly agree). Consistent with the original validation, we calculated a global score as the average across items. Prior studies in Chinese samples have reported good psychometric properties for this scale and its adaptations (e.g., Chen et al., 2024), and our measurement-model assessment confirmed adequate convergent validity, reliability, and discriminant validity in the present context. The full list of items in both English and Chinese is provided in Appendix A.

##### Achievement recognition

Achievement recognition was measured with the adapted version of the Head Behaviors Recognition for Achievement subscale from the Recognition for Job Performance and Achievements questionnaire (Blegen et al., 1992). The 10 items were adapted to the teaching context to capture formal and informal practices through which teachers’ accomplishments are acknowledged (e.g., “The head of department congratulates the teacher in front of peers”). Responses were rated on a 5-point Likert scale ranging from 1 (Not at all) to 5 (Great). Scores were averaged to produce a global index, with higher values indicating stronger perceptions of recognition. Prior organizational research in China has successfully used this instrument and its derivatives to assess recognition processes, reporting satisfactory internal consistency and construct validity. In the present study, the measurement-model assessment further supported the reliability and validity of the adapted scale.

##### Teaching performance

In Study 1, teaching performance was operationalized as task performance, defined as the extent to which teachers effectively fulfill the core duties of their professional role (Motowidlo and Borman, 1997). This variable was measured with the five-item Task Performance subscale of the Individual Work Performance Questionnaire (Koopmans et al., 2014), adapted to the teaching context. Items capture how consistently teachers plan, prioritize, and complete their work (e.g., “I managed to plan my work so that I finished it on time,” “I was able to carry out my work efficiently”). Responses were given on a 5-point Likert scale ranging from 1 (Never) to 5 (Daily). Higher scores indicate greater task-based teaching performance.

##### Time orientation

In Study 1, time orientation was measured with the Chinese 10-item version of the Future Time Perspective Scale (Carstensen et al., 1999). Items assess the extent to which individuals perceive their future as expansive and filled with opportunities or limited and constrained (e.g., “I have the sense that time is running out” [reverse scored]). Responses were rated on a 5-point Likert scale ranging from 1 (Strongly disagree) to 5 (Strongly agree). Scores were averaged to create a global index, with higher values indicating a broader and more opportunity-focused future orientation (Chu and Carstensen, 2023).

#### Data analysis

We tested a first-stage moderated mediation using PROCESS v4.2 for SPSS (Model 7). Professional Growth predicted Achievement Recognition (a-path), which in turn predicted Teaching Performance (b-path), and Future Time Perspective was specified as a moderator of the a-path. Indirect effects were estimated with 5,000 bootstrap resamples. The index of moderated mediation and the conditional indirect effects at W = M, ±1 SD were considered significant when the 95% bias-corrected confidence interval excluded zero.

Because PROCESS (Hayes, 2022) relies on ordinary least squares regression and does not estimate latent variables or provide confirmatory factor analysis (CFA) indices, the measurement model could not be evaluated within the PROCESS framework. To ensure that the adapted instruments met psychometric standards, we conducted an independent measurement-model assessment using PLS-SEM. This approach allowed us to examine convergent validity (AVE), internal consistency (Cronbach’s alpha, rho_A, and composite reliability), discriminant validity (HTMT), and global model fit indices (SRMR, d_ULS, d_G), which are appropriate for variance-based SEM. The structural results reported in Study 1 are therefore based on PROCESS, whereas the measurement-model evaluation was performed separately using PLS-SEM.

#### Measurement model assessment (Study 1)

To evaluate the psychometric properties of the adapted instruments in Study 1, we conducted an independent measurement-model assessment using PLS-SEM, given that PROCESS does not estimate latent variables or provide the necessary indices for assessing measurement quality. Convergent validity was established for all constructs, as their Average Variance Extracted (AVE) values met or exceeded the recommended 0.50 threshold. Specifically, the AVE values were 0.525 for Achievement Recognition, 0.501 for Future Time Perspective, 0.500 for Professional Growth, and 0.631 for Teaching Performance.

Internal consistency was excellent across all measures. Composite reliability ranged from 0.895 to 0.944, rho_A from 0.863 to 0.939, and Cronbach’s alpha from 0.855 to 0.937, all well above commonly accepted cut-off values. Discriminant validity was supported using the heterotrait–monotrait ratio (HTMT), with values ranging from 0.247 to 0.624, comfortably below the conservative 0.85 criterion. The corresponding bootstrapped confidence intervals did not approach problematic levels, further confirming discriminant validity.

Global model fit was examined using PLS-SEM indices. The standardized root mean square residual (SRMR) indicated excellent fit (0.049). Additional global fit statistics, including d_ULS (2.18) and d_G (0.69), fell within acceptable bootstrapped confidence intervals, suggesting adequate correspondence between the empirical and model-implied covariance matrices. Because PLS-SEM does not compute covariance-based CFA indices such as CFI, TLI, or RMSEA, model fit assessment relies on these PLS-specific indices.

A complete summary of convergent validity, reliability estimates, discriminant validity, and model fit indices is provided in the Supplementary Tables S1–S3.

As an additional methodological check, we conducted a brief assessment of potential common method variance (CMV) using Harman’s single-factor test. An exploratory factor analysis of all Study 1 items showed that the first unrotated factor accounted for 28.6% of the total variance, indicating that no single factor dominated the item covariance structure. Although this test cannot eliminate CMV concerns entirely, the results suggest that substantial method inflation is unlikely.

### Results

#### Descriptive statistics and correlations

Before testing our model, a correlation analysis was conducted among the study variables. These results are reported in Table 1. Pearson’s correlations indicated that all significant relationships between the variables were in the expected direction.

**Table 1:** Means, standard deviations, Cronbach’s alphas (on the diagonal), and correlations among all study variables.

Variables	*M*	SD	1	2	3	4
1. Professional growth	3.84	0.77				
2. Achievement recognition	2.31	0.78	0.311**			
3. Future time perspective	3.34	0.63	0.369**	0.227**		
4. Teaching performance (task performance)	3.10	0.70	0.557**	0.285**	0.260**	

N = 234; *p < 0.05; **p < 0.01; ***p < 0.001. Study 1: Teaching Performance was operationalized as task performance (Koopmans et al., 2014).

#### Direct and mediation effects (hypotheses 1 and 2)

In our analysis, we examined both the direct relationship between Professional Growth (X) and Teaching Performance (Y) and the mediating role of Achievement Recognition (M). The results indicated a positive but no significant association between Professional Growth (X) and Achievement Recognition (M), with *B* = 0.146, SE = 0.260, *t* = 0.56, *p* = 0.575, and a 95% CI of [−0.366, 0.659], as Table 2 shows. Furthermore, Achievement Recognition (M) showed a positive association with Teaching Performance (Y), evidenced by *B* = 0.1110, SE = 0.0511, *t* = 2.17, *p* = 0.031, and a 95% CI of [0.0103, 0.2117]. In addition, there was a strong direct association between Professional Growth (X) and Teaching Performance (Y), with *B* = 0.4728, SE = 0.0518, *t* = 9.12, *p* < 0.001, and a 95% CI of [0.3707, 0.5749]. The path from Professional Growth to Achievement Recognition was positive but not statistically significant (a-path: *B* = 0.146, SE = 0.260, *t* = 0.56, *p* = 0.575, 95% CI [−0.366, 0.659]). Achievement Recognition, in turn, showed a significant association with Teaching Performance (b-path: *B* = 0.111, SE = 0.051, *t* = 2.17, *p* = 0.031, 95% CI [0.010, 0.212]). The direct effect of Professional Growth on Teaching Performance remained strong (c′-path: *B* = 0.473, SE = 0.052, *t* = 9.12, *p* < 0.001, 95% CI [0.371, 0.575]). The bootstrapped indirect effect was small but statistically significant (indirect effect = 0.030, boot SE ≈ 0.015, 95% bias-corrected CI [0.004, 0.062]). Thus, a modest indirect association was observed despite uncertainty in the a-path, indicating that Achievement Recognition accounted for a limited portion of the overall association between Professional Growth and Teaching Performance. Standardized coefficients are not provided by PROCESS for models that include interaction terms (Hayes, 2022), so we report unstandardized estimates together with their 95% confidence intervals, in line with current recommendations for moderated mediation models. Accordingly, the results are best interpreted as evidence of a strong direct association accompanied by a weak and secondary indirect effect, rather than as support for a substantive mediation process.

**Table 2:** Model summary and direct effects.

Outcome variable: achievement recognition (M)	*B*	SE	*t*	*p*	95% CI [LL, UL]	*R* ^2^	*F* (d.f.)	*p* (F)
Constant	1.1835	0.9671	1.22	0.222	[−0.7220, 3.0891]	0.112	9.67 (3, 230)	< 0.001
Professional Growth (X)	0.1461	0.2601	0.56	0.575	[−0.3663, 0.6585]			
Future Time Perspective (W)	0.0261	0.2963	0.09	0.930	[−0.5577, 0.6098]			
Interaction (X × W)	0.0367	0.0766	0.48	0.633	[−0.1143, 0.1877]			

Outcome variable: teaching performance (task performance) (Y)	*B*	SE	*t*	*p*	95% CI [LL, UL]	*R* ^2^	*F* (d.f.)	*p* (F)
Constant	2.8474	0.1242	22.92	<0.001	[2.6027, 3.0922]	0.324	55.46 (2, 231)	<0.001
Professional Growth (X)	0.4728	0.0518	9.12	< 0.001	[0.3707, 0.5749]			
Achievement Recognition (M)	0.1110	0.0511	2.17	0.031	[0.0103, 0.2117]			

CI = Confidence Interval, B = Unstandardized Regression Coefficient, SE = Standard Error, d.f. = degrees of freedom. Study 1: Teaching Performance was operationalized as task performance (Koopmans et al., 2014).

#### Effect sizes

The regression predicting Achievement Recognition from Professional Growth, Future Time Perspective, and their interaction explained 11.2% of the variance (*R*^2^ = 0.11; *f*^2^ = 0.13, small–to–medium). The regression predicting Teaching Performance from Professional Growth and Achievement Recognition explained 32.4% of the variance (*R*^2^ = 0.32; *f*^2^ = 0.48, large). For individual predictors, partial effect sizes were as follows: Professional Growth → Recognition (partial *r*^2^ = 0.001; *f*^2^ ≈ 0.00, negligible), Future Time Perspective → Recognition (partial *r*^2^ = 0.017; *f*^2^ = 0.02), Interaction term (partial *r*^2^ = 0.001; *f*^2^ ≈ 0.00), Professional Growth → Teaching Performance (partial *r*^2^ = 0.265; *f*^2^ = 0.36, large), and Achievement Recognition → Teaching Performance (partial *r*^2^ = 0.020; *f*^2^ = 0.02, small).

#### Moderated mediation analysis (hypothesis 3)

Our analysis explored the potential moderating role of Future Time Perspective (W) in the relationship between Professional Growth (X) and Achievement Recognition (M). The aggregate model predicting Achievement Recognition was significant, *F*(3, 230) = 9.67, *p* < 0.001, and *R*^2^ = 0.112. In the moderated mediation model, the interaction between Professional Growth and Future Time Perspective on Achievement Recognition was not significant (B = 0.0367, SE = 0.0766, *p* = 0.633), and the index of moderated mediation was not different from zero (index = 0.0041, SE = 0.0085, 95% CI [−0.0130, 0.0229]). Conditional indirect effects via Achievement Recognition were positive and of similar magnitude at low (−1 SD), mean, and high (+1 SD) levels of Future Time Perspective, indicating no moderation of the a-path. These findings indicate that Future Time Perspective did not moderate the mediating effect of Achievement Recognition, thus Hypothesis 3 was not supported (see Table 3).

**Table 3:** Conditional indirect effects and moderated mediation (testing hypotheses 2 and 3).

Conditional indirect effects of professional growth on teaching performance	Future time perspective	Effect	Bootstrapped SE	Boot 95% CI [LL, UL]
Professional growth → Achievement recognition → Teaching performance	–1 SD (−0.6317)	0.0273	0.0134	[0.0036, 0.0568]
Professional growth → Achievement recognition → Teaching performance	Mean (0.0000)	0.0298	0.0145	[0.0043, 0.0622]
Professional growth → Achievement recognition → Teaching performance	+1 SD (0.6317)	0.0324	0.0172	[0.0032, 0.0718]

Index of moderated mediation	Effect	Bootstrapped SE	Boot 95% CI [LL, UL]
Interaction between professional growth and future time perspective	0.0041	0.0085	[−0.0124, 0.0228]

Effect sizes are unstandardized coefficients. CI = Confidence Interval; SE = Standard Error. Study 1: Teaching Performance was operationalized as task performance (Koopmans et al., 2014).

**Figure 2:**
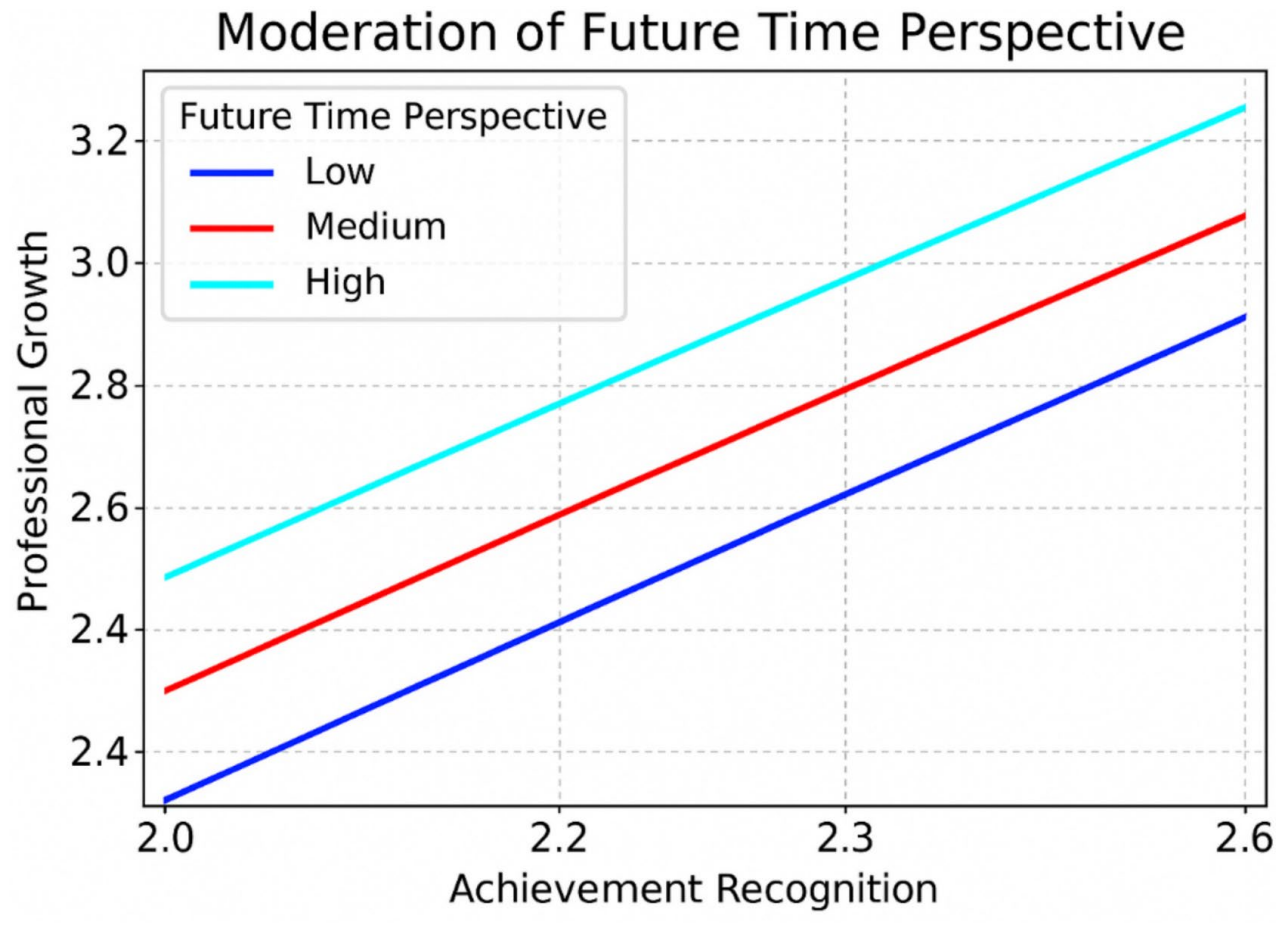
Relationship between professional growth and achievement recognition at low, mean, and high levels of future time perspective. Slopes are positive and similar across levels of the moderator.

Figure 2 displays the non-significant moderating effect of Future Time Perspective on the relationship between Professional Growth and Achievement Recognition, which subsequently affects Teaching Performance.

The plot shows that as Achievement Recognition increases, Professional Growth also increases across all levels of Future Time Perspective, which is evident from the positive slopes of the lines. However, the difference in the steepness of the lines (representing low, medium, and high Future Time Perspective) is relatively minimal. The hypothesis suggested that Future Time Perspective would play a significant role in strengthening the relationship between Professional Growth and Achievement Recognition, particularly when it is high.

**Figure 3:**
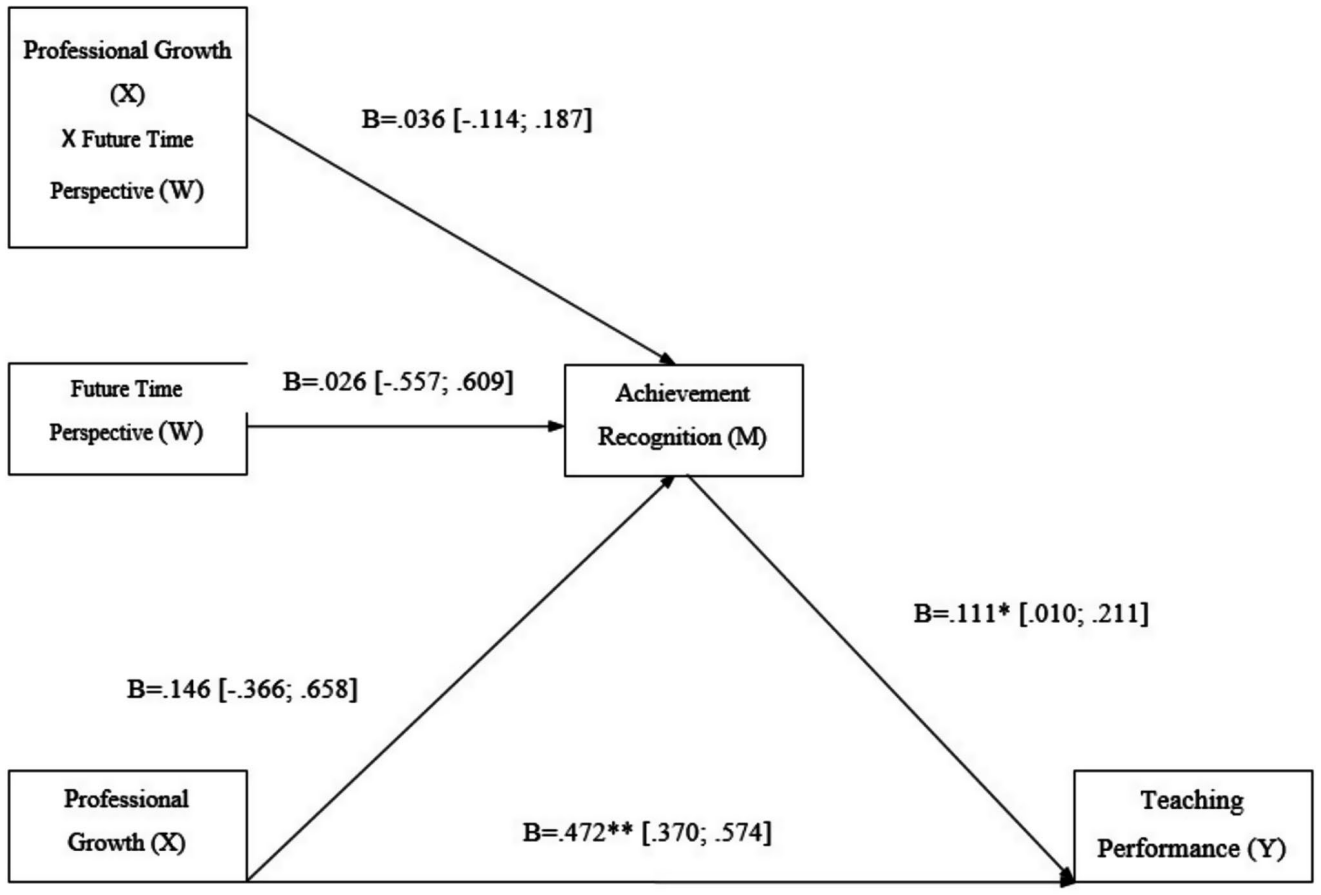
Moderated mediation results. The indirect effect of Professional Growth on Teaching Performance through Achievement Recognition did not vary by Future Time Perspective. [95% CI]; *p < 0.05; **p < 0.01; ***p < 0.001.

In this plot, however, the relationship between Professional Growth and Achievement Recognition appears to be relatively similar across the different levels of Future Time Perspective. While all groups show a positive relationship, the fact that the slopes are similar suggests that Future Time Perspective may not significantly moderate the relationship. This aligns with the statistical findings where the interaction effect was not significant, indicating that Future Time Perspective does not strongly influence how Professional Growth relates to Achievement Recognition. Thus, contrary to Hypothesis 3’s expectation, the relationship between Professional Growth and Teaching Performance (through Achievement Recognition) is not notably stronger for individuals with higher Future Time Perspective based on this visual representation (Figure 3).

### Discussion of Study 1

The findings from Study 1 suggest that Professional Growth was strongly associated with Teaching Performance, and a small but statistically significant indirect effect via Achievement Recognition emerged in the bootstrap analysis. Importantly, however, the a-path from Professional Growth to Achievement Recognition was not significant. This pattern indicates that the indirect effect should be interpreted cautiously and does not support the use of the term “partial mediation.” Small indirect effects may arise even when one constituent path is weak or uncertain, a pattern that can reflect measurement error, limited statistical power for detecting the a-path, or partial suppression effects within the model. From a practical standpoint, the contribution of Achievement Recognition to the link between Professional Growth and Teaching Performance therefore appears modest rather than substantial. Consistent with this interpretation, we describe the results as a weak indirect effect present despite a-path uncertainty, rather than as a robust mediating process. This conceptualization is adopted consistently throughout the manuscript to avoid overinterpreting the indirect association as a robust mediating mechanism.

By contrast, Future Time Perspective did not moderate the a-path, and the index of moderated mediation was not different from zero, indicating that the mediation remained statistically stable across levels of Future Time Perspective. Consistent with these findings, recent work on school leadership and teacher development in China confirms that professional growth is strongly related to teacher self-efficacy and classroom effectiveness (Zhang et al., 2025). Overall, these results indicate that, at least in this context, Future Time Perspective played a limited role as a moderator. FTP did not alter the strength of the pathways linking growth, recognition, and teaching performance, suggesting that temporal orientation may operate more at a mean-level motivational level than as a boundary condition in structural associations.

In addition to these statistical considerations, sociocultural factors may also help explain the weak association between professional growth and recognition. In many Chinese secondary schools, recognition practices tend to be formal, hierarchical, and tied to collective norms, which can lead teachers to experience professional development and recognition as parallel rather than interconnected processes. Teachers may invest in growth for purposes related to self-improvement, compliance, or career progression without necessarily expecting that such efforts will be personally acknowledged by school leaders. This structural separation between development and recognition may help account for the absence of a strong a-path in Study 1. Another alternative model also merits consideration: recognition may function as an antecedent rather than a consequence of growth. In contexts where recognition signals institutional trust and professional value, it may stimulate teachers’ willingness to engage in development activities rather than the reverse. This possibility is consistent with analyses of Chinese school culture, in which recognition by leaders plays an important motivational and symbolic role. Although the present design does not allow for the testing of reciprocal or alternative models, acknowledging this possibility offers a more balanced interpretation of the findings.

The mediating role of Achievement Recognition deserves particular attention. When teachers feel that their work is valued and acknowledged, they are more likely to translate growth opportunities into higher levels of performance. Recognition practices foster a sense of belonging and professional commitment, which then enhance motivation and engagement. Studies in both Chinese rural and urban settings have shown that professional honor and recognition are critical factors for teachers’ career decisions and persistence in the profession (Chen et al., 2025). Evidence on transformational leadership in Chinese schools also supports the idea that recognition processes are central to linking growth with effective teaching (Yu and Jang, 2024).

In contrast, Hypothesis 3 was not supported. The moderating role of Future Time Perspective did not reach significance. Although there was a small descriptive trend suggesting stronger indirect effects for teachers with higher levels of Future Time Perspective, these differences were not statistically meaningful. This suggests that the mediating pathway through Achievement Recognition functions relatively consistently across teachers, regardless of whether they adopt a short- or long-term temporal orientation. This is consistent with the broader literature, which shows that Future Time Perspective is related to work motivation and adaptation, but its moderating effects are not always robust (Mohsin et al., 2024).

These findings should be interpreted with caution. The cross-sectional design prevents causal inference, and the reliance on self-report measures may have reduced sensitivity to detect interaction effects. It is also possible that in the context of Chinese secondary education, teachers share similar temporal orientations, leaving little variance for Future Time Perspective to act as a moderator. Nevertheless, the null result is informative. Nevertheless, the null result is informative. It indicates that Professional Growth is strongly linked to Teaching Performance, and that this pathway may include a small indirect component via Achievement Recognition, although this contribution is weak and should be interpreted cautiously, regardless of differences in time perspective. The limitations of Study 1 point to the need for further investigation. Future research should use experimental or quasi-experimental designs to test causal mechanisms more directly. In particular, Study 2 was designed to manipulate time orientation experimentally by comparing short-term, long-term, and control conditions. This approach allows us to examine whether temporal orientation operates at a broader, mean-level by shaping overall levels of professional growth, achievement recognition, and teaching performance, rather than by strengthening the internal mediational structure identified in Study 1. Thus, the evidence from Study 1 not only clarifies the role of Achievement Recognition but also justifies the continuation of this research program with a more rigorous design.

## Study 2

Study 2 used a three-arm randomized design to examine the effects of Time Orientation on Professional Growth, Achievement Recognition, and Teaching Performance. Teachers were assigned to one of two Time Orientation workshops (short-term vs. long-term) or to a control condition that received general career-development materials. We assessed Professional Growth, Achievement Recognition, Teaching Performance, and a manipulation check for Time Orientation to verify condition differences.

In the experimental extension, we sought to test whether shifting teachers’ temporal orientation toward the future would enhance recognition and performance. Although temporal orientation was measured with the global ZTPI-C, the intervention was designed to prime future-oriented thinking. We therefore hypothesized that teachers in the long-term (future-focused) condition would report higher achievement recognition than those in the short-term or control conditions (H4). We further expected that the indirect pathway from professional growth to teaching performance through recognition would be stronger in the long-term condition (H5). Finally, we hypothesized that teachers in the short-term condition would not show significant increases in recognition compared to the control group (H6).

This design provides a more robust test of the theoretical model by actively manipulating time orientation. It therefore addresses the limitations of Study 1, where Future Time Perspective was measured as a naturally occurring trait and did not significantly moderate the mediation process.

### Method

#### Participants

Participants were 253 Chinese secondary school teachers who completed all sessions and the post-intervention survey. Participants ranged in age from 21 to 58 years (*M* = 35.59, SD = 10.19). Of the sample, 146 were male (57.7%) and 107 were female (42.3%). All participants completed the intervention and the subsequent survey, and no cases were excluded due to missing demographic information. The dataset only included age and gender; information on disciplinary background, regional location, school tier, or urban–rural status was not available.

#### Procedure

This study received ethical approval from the Institutional Review Board of the University of Buenos Aires (Approval Number: 2024-5668-PhiLit, granted June 28, 2024). All procedures complied with the ethical standards of the 1964 Declaration of Helsinki and its later amendments.

Teachers were recruited by disseminating study information across educational institutions in different regions of China. Those who expressed interest were asked to complete an electronic informed consent form. Participants were informed about the study’s objectives, procedures, voluntary nature, and the three experimental conditions.

Following consent, participants were randomly assigned by a computer-generated process to one of three conditions: (a) a short-term time orientation intervention, (b) a long-term time orientation intervention, or (c) a control condition in which participants received only general theoretical material on career development and teaching performance. After providing informed consent, participants were randomly assigned in a 1:1:1 ratio to the short-term time orientation, long-term time orientation, or control condition using the Qualtrics randomizer tool. The allocation was fully automated within the survey platform and concealed until the intervention materials were presented, preventing any influence from researchers or participants on group assignment. To ensure data integrity, Qualtrics’ Prevent Ballot Box Stuffing option was activated and assignment logs were stored for verification. Random allocation ensured that any differences in outcomes could be attributed to the interventions rather than pre-existing characteristics.

All sessions were conducted online via a secure video conferencing platform. Detailed instructions about the procedures were provided to each participant prior to their involvement. The content and timeline of each condition’s workshops were described in the Supplementary materials to guarantee transparency and replicability. Each experimental group received a four-session intervention specifically structured to reflect the assigned condition.

After completing the intervention, participants responded to an electronic survey that included validated measures of Professional Growth, Achievement Recognition, and Teaching Performance, as well as a manipulation check to verify that the experimental conditions were perceived as intended. Supplementary materials provide a description of the workshops administered in each experimental condition (see Appendix B).

#### Instruments

##### Professional growth

In Study 2, professional growth was assessed using the Professional Growth Scale originally developed in Chinese and applied in various professional contexts, including nursing (Chen et al., 2024). The scale consists of 15 items that address perceptions of professional ability development, opportunities for promotion, remuneration, and progress toward career goals. For the purposes of the present study, the items were adapted to the teaching profession (e.g., “I set clear professional goals for my teaching career,” “My teaching competence is continuously improving.”). Participants rated each item on a 5-point Likert scale ranging from 1 (Very inconsistent) to 5 (Very consistent). Responses were summed to yield a total score ranging from 15 to 75, with higher scores indicating greater perceived professional growth. As in Study 1, a translation and back-translation procedure was employed to guarantee linguistic and cultural accuracy (Klotz et al., 2023). The full list of items of the scale in both English and Chinese language is provided as Appendix A.

##### Achievement recognition

Achievement recognition was measured with the adapted version of the Head Behaviors Recognition for Achievement subscale from the Recognition for Job Performance and Achievements questionnaire (Blegen et al., 1992), as used in Study 1. The 10 items were adapted to the teaching context to capture formal and informal practices through which teachers’ accomplishments are acknowledged (e.g., “The head of department congratulates the teacher in front of peers”). Responses were rated on a 5-point Likert scale ranging from 1 (Not at all) to 5 (Great). Scores were averaged to produce a global index, with higher values indicating stronger perceptions of recognition.

##### Teaching performance

In Study 2, teaching performance was measured using five items from the Middle School Teachers’ Classroom Teaching Strategy Scale (MSTCTSC; Wu et al., 2019). The original scale contains 23 items, but only a theoretically coherent subset of five items was used here to capture core aspects of teachers’ classroom performance. Responses were provided on a 5-point Likert scale ranging from 1 (Never) to 5 (Always), with higher scores indicating greater teaching performance (Darling-Hammond et al., 2017). In the present study, the five items were summed to create a global index, yielding possible scores from 5 to 25. For ease of interpretation, all descriptive statistics and ANOVA results for teaching performance in Study 2 are reported using these summed scores.

##### Manipulation check: time orientation

Because the workshops were conducted online, we first included a single attention-check item to ensure that participants had been following the session. Only those who correctly identified the assigned focus (coded = 1) were retained for analysis; those who failed this check were excluded. In addition, participants completed a validated measure of temporal orientation, which served as the main manipulation check. Specifically, we used the Chinese short version of the Zimbardo Time Perspective Inventory (ZTPI-C; Li et al., 2023), which contains 25 items reflecting past, present, and future orientations and has shown a factor structure consistent with the original ZTPI. Items were rated on a 15-point Likert scale (1 = strongly disagree to 15 = strongly agree), and responses were summed to create a global temporal-orientation score (range 25–375), with higher values indicating a more expansive and opportunity-focused temporal outlook. In the present study, the mean global ZTPI-C score was 128.16 (SD = 21.53), consistent with moderate to high levels of future-oriented thinking in this scoring system.

#### Data analysis

All analyses were conducted using SPSS v29 with a significance level of *α* = 0.05. To assess the effectiveness of the manipulation, a one-way ANOVA was performed on Time Orientation (ZTPI-C) scores, followed by Tukey *post hoc* comparisons.

Before conducting the experimental analyses, we independently evaluated the measurement properties of the adapted scales used in Study 2. Because the study relies on ANOVA-based comparisons and the instruments were administered in abbreviated form, we assessed reliability and convergent/discriminant validity using PLS-SEM. This complementary analysis allowed us to examine the psychometric adequacy of the adapted measures (Achievement Recognition, Professional Growth, Teaching Performance, and Time Orientation).

Subsequently, a series of one-way ANOVAs was used to test for differences across the three experimental conditions (short-term, long-term, and control) on the main outcomes: achievement recognition, professional growth, and teaching performance. For each dependent variable, condition was entered as the between-subjects factor. Significant omnibus effects were followed by Tukey post hoc tests to examine pairwise differences. Effect sizes were reported using eta squared (η^2^) to quantify the magnitude of group differences.

Prior to analysis, assumptions of normality and homogeneity of variances were examined. Levene’s test confirmed equality of variances for all outcomes. Only participants who passed the attention check were retained in the final dataset, ensuring data integrity and minimizing missing values. Potential covariates such as age, gender, and teaching experience were examined to determine whether they should be included in the main analyses. Because preliminary inspection did not suggest any systematic influence on the dependent variables, no covariates were ultimately entered in the reported analyses. This decision was made to maintain parsimony and interpretability in the model comparisons.

#### Data availability statement

Anonymized data and analysis scripts for Study 2 are publicly available on the Open Science Framework (OSF) at the following link: https://osf.io/cnv5g/?view_only=772488d1fbb4475fb5ea9dbce3c1cd5b.

#### Measurement model assessment (Study 2)

To evaluate the measurement properties of the adapted instruments used in Study 2, we conducted an independent measurement-model assessment using PLS-SEM. This analysis was performed to complement the ANOVA-based experimental design and to provide evidence of the reliability and validity of the measures, given that the adapted instruments were administered in abbreviated form.

Convergent validity showed mixed results across constructs. Achievement Recognition demonstrated adequate convergent validity (AVE = 0.544), whereas Professional Growth presented an AVE slightly below the recommended 0.50 threshold (AVE = 0.392). Teaching Performance (five-item adapted version of the MSTCTSC) and Time Orientation showed low AVE values (0.242 and 0.078, respectively), indicating limited convergence at the latent level for these abbreviated scales.

Internal consistency varied accordingly. Achievement Recognition and Professional Growth demonstrated strong reliability across indicators (Cronbach’s *α* = 0.907 and 0.889; ρc = 0.923 and 0.906, respectively). As expected for brief scales capturing heterogeneous behaviors, the five-item Teaching Performance measure showed low reliability (α = 0.181; ρc = 0.601), and Time Orientation also presented reduced internal consistency (α = 0.482; ρc = 0.619).

Discriminant validity results were generally acceptable for most constructs, although some pairs approached or exceeded conservative HTMT thresholds. Notably, the HTMT estimate for Time Orientation and Teaching Performance reached values close to or above 1.00, suggesting conceptual overlap or limited discriminant precision between the abbreviated forms of these measures.

Taken together, these findings indicate that the abbreviated versions of Achievement Recognition and Professional Growth function adequately at the construct level, whereas the shortened Teaching Performance and Time Orientation measures exhibit restricted convergent validity and internal consistency. These limitations are reported and discussed in the manuscript as they may reduce measurement precision in Study 2. Full measurement model results are provided in the Supplementary Tables S4–S6.

To examine whether common method variance (CMV) might have influenced the results, we also conducted Harman’s single-factor test for Study 2. The first unrotated factor explained 14.3% of the total variance, well below thresholds indicating a dominant method factor. While CMV cannot be ruled out entirely, this pattern suggests that method inflation is unlikely to account for the observed effects in Study 2.

### Results

#### Manipulation check

Because the interventions were delivered online, participants were first screened to ensure that they had been paying attention during the sessions. Only those who correctly answered the attention check (scoring 1 on the manipulation check item) were included in the analyses.

A one-way ANOVA was conducted on the temporal orientation scores to verify the effectiveness of the manipulation. Results indicated a large overall effect of condition, *F*(2, 250) = 121.49, *p* < 0.001, η^2^ = 0.49. As shown in the Table 4, participants in the long-term perspective condition (*M* = 142.04, SD = 22.39) and the short-term perspective condition (*M* = 137.88, SD = 14.87) scored significantly higher than those in the control group (*M* = 109.30, SD = 5.30; both *p*s < 0.001). The difference between the long-term and short-term conditions was not statistically significant (*p* = 0.21).

**Table 4:** Descriptive statistics and ANOVA results for time orientation (manipulation check) by condition (Study 2).

Condition	*n*	*M*	SD	95% CI [LL, UL]
Control group	98	109.30	5.30	[108.23, 110.36]
Short-term perspective	73	137.88	14.87	[134.41, 141.34]
Long-term perspective	82	142.04	22.39	[137.12, 146.96]

ANOVA: *F*(2, 250) = 121.49, *p* < 0.001, η^2^ = 0.49. *Post hoc* tests (Tukey): Control < Short-term, Control < Long-term (both *p* < 0.001); Short-term ≈ Long-term (n.s.). Study 2: Teaching Performance was assessed with the MSTCTSC (Wu et al., 2019).

These findings confirm that the manipulation successfully altered participants’ temporal orientation, with both experimental groups reporting higher orientation scores than the control group.

Although not initially hypothesized, we also examined the Future Orientation subscale of the ZTPI-C as a more targeted, exploratory manipulation check. For participants with complete item-level data (N = 224), a one-way ANOVA on the summed future-orientation scores revealed a significant effect of condition, *F*(2, 221) = 28.41, *p* < 0.001, η^2^ = 0.21. Consistent with the intended focus of the intervention, teachers in the long-term condition reported the highest future orientation (*M* = 27.96, SD = 5.46), followed by those in the short-term condition (*M* = 23.73, SD = 7.42), whereas the control group showed the lowest scores (*M* = 21.30, SD = 2.56). Tukey *post hoc* tests indicated that all pairwise differences were statistically significant (long-term > short-term > control). Because this analysis was conducted post hoc and was not pre-specified in our hypotheses, we interpret these results descriptively, as convergent evidence that the intervention shifted participants’ future-focused thinking in the expected direction. Full descriptive statistics and ANOVA results for the Future Orientation subscale are reported in Supplementary Table S7.

#### Achievement recognition

A one-way ANOVA was conducted to examine differences in achievement recognition across the three experimental conditions. The analysis revealed a significant effect of condition, *F*(2, 250) = 31.54, *p* < 0.001, η^2^ = 0.20. Participants in the long-term orientation condition (*M* = 4.38, SD = 0.65) reported the highest levels of achievement recognition, followed by those in the short-term orientation condition (*M* = 3.95, SD = 0.83), and the control group (*M* = 3.45, SD = 0.85). *Post hoc* comparisons (Tukey) showed that both the long-term and short-term conditions were significantly higher than the control group (both *p* < 0.001), and the long-term condition was also significantly higher than the short-term condition (*p* = 0.002). As shown in Table 5, these findings confirm the hypothesis that temporal orientation interventions increase achievement recognition, with the long-term orientation condition producing the strongest effect.

**Table 5:** Descriptive statistics for achievement recognition by condition (Study 2).

Condition	*n*	*M*	SD	95% CI [LL, UL]
Control group	98	3.45	0.85	[3.28, 3.62]
Short-term orientation	73	3.95	0.83	[3.75, 4.14]
Long-term orientation	82	4.38	0.65	[4.24, 4.52]

ANOVA: *F*(2, 250) = 31.54, *p* < 0.001, η^2^ = 0.20. *Post hoc* tests (Tukey): Long-term > Short-term > Control. Study 2: Teaching Performance was assessed with the MSTCTSC (Wu et al., 2019).

#### Professional growth

A one-way ANOVA was conducted to test for differences in professional growth across the three conditions. The results indicated a strong effect of condition, *F*(2, 250) = 50.04, *p* < 0.001, η^2^ = 0.29. Participants in the long-term orientation condition (*M* = 56.04, SD = 9.32) reported significantly higher levels of professional growth than those in both the control group (*M* = 42.39, SD = 12.30) and the short-term orientation condition (*M* = 37.60, SD = 14.32). In addition, the control group also scored significantly higher than the short-term perspective group (*p* = 0.029).

As shown in Table 6, the pattern of results was clear: the long-term orientation condition showed the highest levels of professional growth, the control condition was in the middle, and the short-term orientation condition showed the lowest levels. These findings are consistent with the hypothesis that temporal orientation is associated with meaningful differences in professional growth, with long-term orientation being especially beneficial.

**Table 6:** Descriptive statistics for professional growth by condition (Study 2).

Condition	*n*	*M*	SD	95% CI [LL, UL]
Control group	98	42.39	12.30	[39.92, 44.85]
Short-term orientation	73	37.60	14.32	[34.26, 40.95]
Long-term orientation	82	56.04	9.32	[53.99, 58.08]

ANOVA: *F*(2, 250) = 50.04, *p* < 0.001, η^2^ = 0.29. *Post hoc* tests (Tukey): Long-term > Control > Short-term. Study 2: Teaching Performance was assessed with the MSTCTSC (Wu et al., 2019).

#### Teaching performance

A one-way ANOVA tested differences in teaching performance across the three conditions. The results showed a significant main effect, *F*(2, 250) = 22.22, *p* < 0.001, η^2^ = 0.15. Participants in the long-term orientation condition (*M* = 15.90, SD = 3.07) reported significantly higher teaching performance than those in both the control group (*M* = 12.71, SD = 2.87, *p* < 0.001) and the short-term orientation condition (*M* = 13.97, SD = 3.73, *p* < 0.001). In addition, the short-term orientation condition scored significantly higher than the control group (*p* = 0.030).

As presented in Table 7, the findings are consistent with the hypothesis that temporal orientation is associated with differences in teaching performance, with the long-term perspective condition showing the strongest positive effect.

**Table 7:** Descriptive statistics for teaching performance by condition (Study 2).

Condition	*n*	*M*	SD	95% CI [LL, UL]
Control group	98	12.71	2.87	[12.13, 13.28]
Short-term orientation	73	13.97	3.73	[13.10, 14.84]
Long-term orientation	82	15.90	3.07	[15.22, 16.57]

ANOVA: *F*(2, 250) = 22.22, *p* < 0.001, η^2^ = 0.15. Post hoc tests (Tukey): Long-term > Short-term > Control. Study 2: Teaching performance scores range from 5 to 25; higher values indicate better performance.

To complement the ANOVA results and η^2^ estimates, we computed Cohen’s *d* for the pairwise comparisons. For Achievement Recognition, effect sizes were small to moderate for Short-term vs. Control (*d* = 0.54, 95% CI [0.21, 0.86]) and moderate for Long-term vs. Short-term (*d* = 0.56, 95% CI [0.23, 0.89]), whereas the contrast between Long-term and Control was large (*d* = 1.15, 95% CI [0.81, 1.50]). For Teaching Performance, effect sizes were small for Short-term vs. Control (*d* = 0.37, 95% CI [0.05, 0.70]), moderate for Long-term vs. Short-term (*d* = 0.59, 95% CI [0.26, 0.92]), and large for Long-term vs. Control (*d* = 1.10, 95% CI [0.76, 1.44]). For Professional Growth, the long-term group showed a very large advantage over both the control (*d* = 1.18, 95% CI [0.83, 1.53]) and the short-term group (*d* = 1.54, 95% CI [1.17, 1.91]), whereas the short-term group scored lower than control (*d* = −0.43, 95% CI [−0.76, −0.11]). Full results are presented in Supplementary Table S8.

#### Hypotheses testing

The results provided strong support for Hypothesis 4. As shown in Table 5, teachers in the long-term orientation condition reported significantly higher levels of Achievement Recognition (*M* = 4.38, SD = 0.65) compared to both the short-term condition (*M* = 3.95, SD = 0.83, *p* = 0.002) and the control group (*M* = 3.45, SD = 0.85, *p* < 0.001). This pattern is consistent with the view that a long-term temporal orientation is especially conducive to higher levels of perceived achievement recognition. In contrast, Hypothesis 5 was not supported. Beyond the ANOVAs showing significant differences between conditions on professional growth (Table 6) and teaching performance (Table 7), we conducted follow-up mediation analyses to estimate the indirect effect of professional growth on teaching performance through achievement recognition separately within each experimental condition (PROCESS Model 4; Hayes, 2022). As summarized in Supplementary Table S9, the association between professional growth and recognition was weak and inconsistent across groups, and the indirect effect was small and not statistically significant in the long-term and short-term conditions, and only weak in the control condition. Thus, there was no evidence that the indirect pathway from professional growth to teaching performance via recognition was stronger in the long-term condition than in the other conditions, and Hypothesis 5 remains unsupported.

Hypothesis 6 was supported. Teachers in the short-term condition did not differ significantly from those in the control group with respect to Achievement Recognition (*p* > 0.05; see Table 6). This indicates that short-term orientation does not provide an advantage over the control condition in terms of recognition.

### Discussion of Study 2

The experimental results provide further evidence for the relevance of temporal orientation in teachers’ professional development. Consistent with Hypothesis 4, the long-term orientation condition was associated with significantly higher levels of achievement recognition compared to both the short-term and control conditions. Importantly, the limited reliability and convergent validity of the abbreviated measures—particularly for teaching performance and temporal orientation—require that the findings be interpreted as indicative of mean-level differences rather than as evidence for fine-grained structural or mediational processes. Although the ZTPI-C captures multiple temporal frames, the intervention was specifically designed to prime future-oriented thinking. The findings therefore suggest that inviting teachers to adopt a longer temporal horizon tends to co-occur with a stronger sense that their contributions are valued, reinforcing the role of recognition in professional life while not, in itself, demonstrating a direct mechanistic effect of temporal orientation on recognition or growth.

By contrast, Hypothesis 5 was not supported. The indirect pathway from professional growth to teaching performance through achievement recognition did not appear more strongly in the long-term condition, and the mediation itself was not consistently observed across the three groups. This suggests that the mechanism identified in Study 1 may not replicate reliably under experimental variation in temporal orientation, at least within the parameters and constraints of the present design. Accordingly, we refrain from making causal mediation claims in Study 2. The findings indicate that temporal orientation is associated with higher mean levels of recognition, growth, and performance, but do not show that recognition mediates the effect of the manipulation on teaching performance.

Finally, the results supported Hypothesis 6, showing that the short-term orientation condition did not differ from the control condition in achievement recognition. One plausible explanation is that a short-term focus is already highly salient in many Chinese secondary schools, where teachers’ daily work is organized around immediate classroom demands, exam preparation, and performance indicators. Under these circumstances, an additional short-term prime may have limited incremental impact, whereas a long-term prime introduces a qualitatively different perspective that highlights career trajectories and future opportunities. Motivationally, teachers who are invited to adopt a long-term temporal horizon may feel more justified in investing effort in growth and in attending to recognition signals, while those primed with a short-term focus remain anchored in immediate tasks. Taken together, these results indicate that a long-term temporal orientation is conditionally associated with higher levels of recognition, growth, and teaching performance, while also underscoring the limits of short-term interventions and the complexity of recognition as a psychosocial process.

## General discussion

The present research examined how professional growth, achievement recognition, and temporal orientation interact to shape teaching performance among Chinese secondary school teachers. Across two complementary studies—a cross-sectional design and an experimental manipulation—we found converging evidence that professional growth is strongly linked to teaching performance, and that achievement recognition provides a small but statistically significant psychosocial pathway in this association. At the same time, the mediational contribution of recognition was modest in size and should be interpreted cautiously rather than as a strong or exhaustive explanation. Temporal orientation did not show the expected moderated mediation pattern, and its influence appears to be more contextual—shaping mean levels of key variables—than structural in terms of altering internal pathways. Throughout the discussion, we therefore prioritize the strong direct association between professional growth and teaching performance, treating recognition as a modest complementary pathway and temporal orientation as a contextual influence rather than a causal mechanism.

By highlighting achievement recognition as one plausible mediator between growth and performance, this research adds a psychosocial layer to existing models of teacher motivation and effectiveness. It integrates constructs traditionally studied in educational administration—such as professional development and recognition—within psychological frameworks of motivation (Self-Determination Theory) and self-efficacy (Social Cognitive Theory), suggesting that teachers’ perceived acknowledgment can satisfy core psychological needs and reinforce the link between growth opportunities and actual performance, even if this mediational contribution is statistically modest.

Study 1 confirmed that teachers who reported greater professional growth also reported higher levels of teaching performance, and that a small but significant indirect effect through achievement recognition was present. This pattern suggests that recognition can act as a psychosocial bridge between growth and performance, although its mediational contribution is limited in magnitude. Growth opportunities alone are not always sufficient to improve outcomes; they may become more effective when teachers feel that their efforts are acknowledged and valued. This aligns with work in the Chinese context showing that recognition practices are critical for sustaining teacher motivation and retention (Kong et al., 2024; Wang et al., 2024), and with evidence that feedback and recognition support the consolidation of professional identity (Liao et al., 2022; Peng and Nair, 2022).

Our findings also complement cross-professional research suggesting that professional growth is not a purely technical process but involves narrative and meaning-making dimensions. Reflective accounts of one’s career trajectory and recognition from others can consolidate a sense of purpose and engagement (Can, 2019; Khoo, 2017). In this light, recognition can be seen as the mechanism through which developmental experiences are integrated into a coherent professional self-concept that supports performance.

By contrast, the moderating role of Future Time Perspective (FTPS) was not supported in Study 1. Although teachers with higher FTP tended to show slightly stronger indirect effects, these differences were not statistically significant. One interpretation is that the temporal outlook of Chinese secondary teachers is relatively homogeneous, shaped by long-term, duty-oriented norms grounded in Confucian values of perseverance and collective responsibility. In such a context, FTP may function more as a background characteristic than as a differentiating moderator of specific performance pathways. Alternatively, FTP may be more relevant for broad indicators of well-being and persistence rather than for the internal structure of growth–recognition–performance links (Guo et al., 2022; Mohsin et al., 2024).

Building upon these correlational findings, Study 2 refined the model through an experimental manipulation of temporal orientation. Whereas Study 1 relied on cross-sectional data to map correlational pathways among growth, recognition, performance, and future time perspective, Study 2 tested whether experimentally shifting teachers’ temporal focus would change mean levels of growth, recognition, and performance. The long-term condition produced higher levels of achievement recognition, professional growth, and teaching performance than the control condition, indicating that fostering a broader temporal horizon can elevate these outcomes at the mean level. This pattern is consistent with evidence that participation in long-term career planning and appraisal systems enhances teachers’ sense of recognition and organizational belonging (Peng and Nair, 2022). However, the hypothesized strengthening of the indirect pathway from growth to performance through recognition (H5) was not supported. The mediational pattern observed in Study 1 did not replicate consistently across the three experimental conditions, suggesting that recognition processes operate as a relatively stable psychosocial mechanism that is not easily modified through short-term shifts in temporal orientation. In line with this, we interpret the Study 2 findings as conditional associations rather than evidence of a causal mediation process.

Taken together, the two studies contribute to a more nuanced understanding of professional development in Chinese secondary education. Professional growth consistently predicts teaching performance, and recognition emerges as a consistent, though modest, pathway linking these constructs, whereas the influence of temporal orientation appears indirect and contextual. Long-term perspectives may elevate overall recognition and related outcomes, yet they do not fundamentally change the internal mediation process observed in Study 1. This integration of correlational and experimental evidence strengthens a cautious psychological interpretation of the findings, suggesting that recognition may operate as a relatively stable motivational mechanism across methods and designs, while falling short of a strong mediating effect. It also aligns with structural analyses of teacher development in China (Kong et al., 2024), which suggest that institutionalized career ladders and teaching–research groups already embed a long-term orientation into teachers’ professional routines. In such systems, interventions aimed at enhancing temporal orientation may yield incremental rather than transformative gains.

These conclusions have important implications for policy and practice. For school leaders and policymakers, investing in professional growth is necessary but insufficient; it must be accompanied by recognition systems that visibly value teachers’ efforts. Recognition practices—such as formal awards, public acknowledgment, and supportive supervision—are especially crucial in environments where professional advancement is tightly regulated and competition is high (Chen et al., 2024; Peng and Nair, 2022). In addition, programs that promote reflective professional narratives and peer-based recognition may help sustain teachers’ sense of meaning and continuity over time (Khoo, 2017).

In summary, this research highlights a triad of factors that support teaching performance in the Chinese secondary context: opportunities for professional growth, recognition of achievements, and a future-oriented perspective that shapes the broader motivational climate. Within this triad, recognition emerges as a central psychosocial mechanism linking growth to performance, albeit with a statistically modest mediating contribution, while temporal orientation appears to operate primarily as a contextual enhancer rather than as a moderator of internal pathways. Supporting teachers in these dimensions is not only a matter of organizational policy but also a way of sustaining the conditions under which effective teaching can be maintained over time Perry (2023).

### Limitations of the present research and suggestion for future studies

Although the present research offers new insights into the relationships between professional growth, achievement recognition, temporal orientation, and teaching performance, several limitations should be noted. First, Study 1 relied on a cross-sectional design, which prevents strong causal inference. While Study 2 introduced an experimental manipulation to address this limitation, its online format may have reduced ecological validity. Future studies would benefit from longitudinal and multi-method designs that can more accurately capture the dynamic interplay of growth, recognition, and temporal orientation across the teaching career.

Second, both studies used self-report instruments, which raises concerns about common method variance (CMV) and social desirability bias. We conducted Harman’s single-factor tests in both studies, and the first unrotated factor accounted for 28.6 and 14.3% of the total variance, respectively, suggesting that no single method factor dominated the item covariance structure. Nevertheless, because all substantive variables were measured at the same time and from the same source, some degree of method-related inflation cannot be ruled out. Future research should therefore combine procedural and statistical remedies—such as temporal separation of measures, inclusion of marker variables, and confirmatory factor tests for CMV—with multi-source assessments (e.g., classroom observations, peer or administrator ratings, and student outcomes) to reduce reliance on teachers’ subjective perceptions and better isolate true score relationships.

A further limitation concerns the measurement properties of the abbreviated scales used in Study 2. Although the five-item versions of Achievement Recognition and Professional Growth demonstrated good internal consistency, the shortened measures of Teaching Performance and Time Orientation showed limited convergent validity and reduced reliability in the independent measurement-model assessment conducted with PLS-SEM. These indices are likely a consequence of the abbreviated nature of the scales, which capture broad and heterogeneous teaching-related behaviors with a small number of items. As such, effects observed in Study 2 should be interpreted with caution, as measurement precision may have been reduced. Future work would benefit from administering the full validated versions of these instruments or from modeling the constructs at the dimensional level.

Third, the research focused exclusively on secondary school teachers in China. While this context is particularly relevant due to its institutionalized structures for professional growth and cultural emphasis on teacher honor, it also limits the generalizability of findings (Rice, 2024). Future research should extend the model to other educational levels and cultural settings (Chryssouli and Koutroukis, 2023), examining whether the mediating role of recognition and the influence of temporal orientation operate similarly in different systems.

Fourth, the datasets for both studies lacked contextual descriptors that would enable a more fine-grained characterization of the sample (e.g., disciplinary field, regional distribution, or school-level indicators such as urban–rural status and school tier). Although Study 1 included information about age, gender, teaching experience, and role, no further stratification variables were available, and Study 2 included only age and gender. This absence limits the degree to which readers can evaluate generalizability across different types of schools or teacher subgroups. Future research would benefit from collecting richer contextual information and implementing stratified recruitment procedures to better capture heterogeneity across educational settings.

Finally, the findings regarding temporal orientation were less conclusive than expected. In Study 1, the specific measure of future time perspective showed no robust moderating effects. In Study 2, the use of the ZTPI-C provided a broader measure of time perspective, but this may have diluted effects specific to future orientation. Future studies might explore alternative operationalizations of temporal perspective, investigate its longitudinal stability, or examine whether it interacts with other psychosocial resources such as resilience or organizational trust (Nöhammer, 2024). Moreover, the present research did not include psychological outcomes such as motivation, engagement, or well-being, which are theoretically linked to recognition and temporal orientation. Incorporating such measures could help clarify the broader motivational mechanisms through which professional growth translates into performance and satisfaction.

In sum, while the present research clarifies how growth, recognition, and temporal outlook relate to teaching performance, more work is needed to establish causal mechanisms, diversify measurement strategies, and test generalizability across settings. Addressing these directions will not only refine theoretical models but also deepen understanding of how teachers can be supported in their careers in ways that sustain both their professional development and their impact on students.

### Implications for teachers, principals and educational organizations

Given the mixed and partly modest nature of some findings—particularly regarding mediation and temporal orientation—the implications below should be interpreted as tentative directions rather than definitive prescriptions, with stronger emphasis placed on the more consistent associations between growth, recognition, and performance. The findings of this research carry several important implications for teachers, principals, and educational organizations. For teachers, the evidence highlights the value of seeking opportunities for professional growth while also recognizing the importance of having their efforts acknowledged. Growth alone can expand skills and knowledge, but it is achievement recognition—both formal and informal—that transforms those experiences into motivation and sustained performance. Teachers may therefore benefit from actively engaging in feedback processes, sharing their successes with colleagues, and cultivating long-term perspectives that help them see their careers as meaningful trajectories rather than isolated tasks. In applied psychological terms, such practices can be framed as self-regulatory and motivational strategies that enhance perceived competence and autonomy, aligning with evidence that structured coaching and reflective professional dialogue improve teachers’ instructional beliefs and skills (Hu et al., 2022).

For principals, the results emphasize the critical role of recognition practices in school leadership. While professional development opportunities are often mandated at the system level, the daily culture of acknowledgment is shaped locally. Principals can create environments where teachers feel valued by offering constructive feedback, celebrating achievements, and fostering a climate of respect. These practices not only strengthen the link between growth and performance but also enhance teachers’ sense of belonging, reducing turnover intentions and improving morale. Leadership programs that incorporate mentoring or long-term goal-setting components could further reinforce these outcomes, encouraging teachers to internalize recognition as part of their ongoing professional identity (Ma et al., 2024). Encouraging conversations about career planning and broader professional horizons may also help maintain teachers’ motivation over time.

For educational organizations and policymakers, the research underscores the need to design professional development systems that integrate growth opportunities with recognition structures and career planning. Programs should avoid treating training as a purely technical exercise and instead connect it with mechanisms that highlight teachers’ accomplishments and reinforce their professional honor. Psychoeducational interventions that combine coaching, feedback, and long-term goal framing can be especially effective, as they not only enhance teaching competence but also address teachers’ motivational needs and sense of self-efficacy. Interventions that promote long-term temporal orientations—such as mentoring programs, structured career ladders, or vision-building workshops—can further support teachers in envisioning their futures within the profession. At the organizational level, balancing accountability with autonomy remains essential: recognition must be genuine, not just procedural, to sustain motivation and effectiveness.

Taken together, these implications suggest that supporting teachers’ performance requires a holistic approach that considers growth, recognition, and temporal orientation in tandem. When teachers feel that their professional development is acknowledged and connected to a broader career horizon, they are more likely to remain engaged, resilient, and effective. For schools and systems, investing in this triad is not merely a strategy for improving performance metrics, but a commitment to fostering a profession that sustains those who dedicate their lives to shaping future generations.

## Conclusion

This research set out to examine how professional growth, achievement recognition, and temporal orientation interact to shape teaching performance among secondary school teachers in China. By combining a cross-sectional study and an experimental design, we were able to identify both consistent patterns and important nuances. Across both studies, professional growth emerged as a robust predictor of teaching performance, and achievement recognition contributed a small but reliable indirect pathway that helps to explain, in part, why growth opportunities are associated with better performance. Recognition thus appears as an important psychosocial element in the teaching life cycle, reminding us that teachers’ professional development is more likely to translate into effective practice when their efforts are visibly acknowledged.

At the same time, the role of temporal orientation proved more complex. In Study 1, future time perspective did not moderate the pathway from growth through recognition to performance, suggesting that recognition processes operate relatively independently of temporal outlook. In Study 2, experimental manipulation of temporal orientation showed that encouraging long-term perspectives can strengthen perceptions of recognition, though it did not fundamentally alter the mediating process. These findings suggest that temporal framing may enhance the climate of recognition, but recognition itself remains a stable driver that warrants direct attention in policy and practice.

Taken together, the evidence highlights a triad of resources that sustain teachers’ motivation and effectiveness: professional growth, achievement recognition, and the ability to envision a meaningful future. Supporting teachers in these dimensions requires more than technical training; it calls for systems that honor teachers’ contributions and cultivate a horizon of long-term purpose. In this way, the study contributes not only to academic debates on teacher development but also to the lived reality of schools, where teachers’ professional journeys profoundly shape the learning and futures of the students they serve.

## Data Availability

The datasets presented in this study can be found in online repositories. The names of the repository/repositories and accession number(s) can be found below: anonymized data and analysis scripts for Study 1 and 2 are publicly available on the Open Science Framework (OSF) at the following link: https://osf.io/cnv5g/?view_only=772488d1fbb4475fb5ea9dbce3c1cd5b.
