# Supplementary material for: Temporal orientation and the teaching life cycle: pathways linking teachers’ growth, recognition, and performance across two studies
Source: Front Psychol. 2026 Jan 9;16:1720811. doi: 10.3389/fpsyg.2025.1720811 (PMC12827188; doi:10.3389/fpsyg.2025.1720811)
Supplement: Supplementary file 1 [file Data_Sheet_1.docx]

**Appendix A. Professional Growth scales for both studies.**

Study 1. Professional Growth (adapted from Career Growth Scale for Nurses (Ni et al., 2023)

| **Item** | **English version (adapted to teaching)** | | **Chinese version (adapted)** |
| --- | --- | --- | --- |
| Study 1 | | | |
| 1 | | My sense of responsibility as a teacher is getting stronger and stronger. | 我作为教师的责任感越来越强。 |
| 2 | | I gradually realize my role as a teacher and can better adapt to it. | 我逐渐认识到自己作为教师的角色，并能更好地适应它。 |
| 3 | | I increasingly realize the importance of teaching. | 我越来越认识到教学的重要性。 |
| 4 | | My communication with students and colleagues is continuously improving. | 我与学生和同事的沟通能力不断提高。 |
| 5 | | I am increasingly able to consider situations from my students’ perspectives. | 我越来越能够从学生的角度考虑问题。 |
| 6 | | My teaching competence is improving. | 我的教学能力正在提高。 |
| 7 | | My ability to manage my emotions in the classroom is improving. | 我在课堂上管理情绪的能力正在提高。 |
| 8 | | I am taking on more important tasks and responsibilities at my school. | 我在学校承担越来越重要的任务和责任。 |
| 9 | | I am upgrading my academic qualifications or pursuing further education relevant to teaching. | 我正在提升学术资历或攻读与教学相关的进一步教育。 |
| 10 | | My career advancement as a teacher is progressing on schedule. | 我的教师职业晋升按计划进行。 |
| 11 | | My role as a teacher is expanding, such as taking leadership or mentoring positions. | 我作为教师的角色正在扩大，例如担任领导或指导职位。 |
| 12 | | My salary or benefits are improving compared to earlier stages of my career. | 与职业早期相比，我的薪资或福利正在改善。 |
| 13 | | My prestige as a teacher is increasing because of my professional abilities and achievements. | 由于我的专业能力和成就，我作为教师的声望正在提高。 |
| 14 | | I am able to set clear career goals as a teacher. | 我能够设定清晰的教师职业目标。 |
| 15 | | I am able to make concrete plans to achieve my professional goals. | 我能够制定具体计划来实现我的职业目标。 |
| 16 | | My efforts as a teacher bring me closer to achieving my professional goals. | 我作为教师的努力让我更接近职业目标。 |
| 17 | | I carry out my career development plans conscientiously. | 我认真地执行我的职业发展计划。 |

Study 2. Professional Growth originally developed in Chinese and applied in various professional contexts, including nursing (Chen et al., 2024).

| **Item** | **English version (adapted to teaching)** | **Chinese version (adapted)** |
| --- | --- | --- |
| Study 2 | | |
| 1 | I set clear professional goals for my teaching career. | 我为我的教师职业设定了清晰的目标。 |
| 2 | I make practical plans to achieve my career goals. | 我制定切实可行的计划来实现职业目标。 |
| 3 | My efforts as a teacher bring me closer to achieving my goals. | 我作为教师的努力让我更接近实现目标。 |
| 4 | I carry out my professional plans conscientiously. | 我认真执行我的职业计划。 |
| 5 | My teaching competence is continuously improving. | 我的教学能力在不断提高。 |
| 6 | My responsibility and commitment to teaching are getting stronger. | 我对教学的责任感和投入度越来越强。 |
| 7 | My communication and coordination skills as a teacher are improving. | 我作为教师的沟通和协调能力正在提高。 |
| 8 | I am taking on more important tasks at my school. | 我在学校承担越来越重要的任务。 |
| 9 | I am promoted in a timely manner according to the school’s promotion ladder. | 我根据学校的晋升机制及时得到晋升。 |
| 10 | My role as a teacher is expanding (e.g., leadership, mentoring). | 我作为教师的角色正在扩大（例如担任领导或指导）。 |
| 11 | My salary or benefits are improving in my current school. | 我在目前学校的薪资或福利正在提高。 |
| 12 | My prestige as a teacher is increasing due to my achievements. | 由于我的成就，我作为教师的声望正在提高。 |
| 13 | I am recognized for my educational qualifications or further studies. | 我的教育资历或进修学习得到认可。 |
| 14 | I am entrusted with more important responsibilities over time. | 随着时间的推移，我被赋予更重要的职责。 |
| 15 | I feel that my career development as a teacher is progressing steadily. | 我觉得作为教师的职业发展在稳步推进。 |

**Appendix B: Detailed Session-by-Session Script for the Temporal-Orientation Intervention**

Session-by-Session Outline

Session 1.

For the short-term orientation group, the first session introduced participants to short-term goal-setting techniques and immediate planning strategies. Each participant was guided through activities focusing on goals achievable within the next few weeks. In contrast, the long-term orientation group began by exploring long-term career aspirations and developing plans spanning a year or more. The control group attended a parallel session that covered general educational development topics, unrelated to time perspective.

Sessions 2–4.

The subsequent sessions for the short-term group included incremental goal refinement and short-term achievement tracking, while the long-term group engaged in deeper exploration of long-range professional milestones and extended planning. The control group continued to receive general theoretical content on teaching performance and career development.

Session 5–6.

All groups completed reflective and integrative activities aligned with their assigned temporal frame. The short-term group consolidated immediate goals, the long-term group elaborated multi-year professional narratives, and the control group engaged in neutral reflections on teaching practices without temporal framing.

All sessions were delivered online, and each activity was carefully documented to ensure clarity and replicability.

This session-by-session script corresponds to the full set of prompts and activities used in the intervention and is provided to ensure full reproducibility of Study 2.

**Supplemental Material.**

**Table S1. Convergent Validity and Reliability Indicators (Study 1)**

| **Construct** | **AVE** | **Composite Reliability (ρc)** | **Rho_A** | **Cronbach’s α** |
| --- | --- | --- | --- | --- |
| Achievement Recognition | 0.525 | 0.917 | 0.905 | 0.899 |
| Future Time Perspective | 0.501 | 0.909 | 0.896 | 0.891 |
| Professional Growth | 0.500 | 0.944 | 0.939 | 0.937 |
| Teaching Performance | 0.631 | 0.895 | 0.863 | 0.855 |

*Note. AVE ≥ .50 indicates adequate convergent validity. Acceptable reliability is reflected by composite reliability, rho_A, and Cronbach’s alpha values ≥ .70.*

**Table S2. Discriminant Validity (HTMT) With 95% Bootstrapped Confidence Intervals (Study 1)**

| **Construct Pair** | **HTMT** | **95% CI LL** | **95% CI UL** |
| --- | --- | --- | --- |
| Future Time Perspective ↔ Achievement Recognition | 0.247 | 0.164 | 0.381 |
| Professional Growth ↔ Achievement Recognition | 0.324 | 0.216 | 0.432 |
| Professional Growth ↔ Future Time Perspective | 0.420 | 0.252 | 0.577 |
| Teaching Performance ↔ Achievement Recognition | 0.315 | 0.202 | 0.443 |
| Teaching Performance ↔ Future Time Perspective | 0.311 | 0.187 | 0.451 |
| Teaching Performance ↔ Professional Growth | 0.624 | 0.539 | 0.708 |

*Note. All HTMT values are below the conservative .85 threshold, supporting discriminant validity.*

**Table S3. Global Model Fit Indices (PLS-SEM) for Study 1**

| **Index** | **Saturated Model** | **Estimated Model** | **95% CI LL** | **95% CI UL** |
| --- | --- | --- | --- | --- |
| SRMR | 0.049 | 0.049 | 0.054 | 0.057 |
| d_ULS | 2.157 | 2.179 | 2.659 | 2.929 |
| d_G | 0.692 | 0.692 | 0.917 | 0.991 |

*Note. SRMR < .08 indicates good model fit; values < .05 indicate excellent fit. d_ULS and d_G values fell within acceptable bootstrapped confidence intervals.*

**Table S4. Convergent Validity and Reliability Indicators (Study 2)**

| **Construct** | **AVE** | **Composite Reliability (ρc)** | **Rho_A** | **Cronbach’s α** |
| --- | --- | --- | --- | --- |
| Achievement Recognition | 0.544 | 0.923 | 0.907 | 0.907 |
| Professional Growth | 0.392 | 0.906 | 0.892 | 0.889 |
| Teaching Performance | 0.242 | 0.601 | 0.206 | 0.181 |
| Time Orientation | 0.078 | 0.619 | 0.501 | 0.482 |

*Note. AVE ≥ .50 indicates adequate convergent validity; reliability ≥ .70 is considered acceptable. Lower AVE and reliability values for Teaching Performance and Time Orientation reflect the limitations of abbreviated scales.*

**Table S5. Discriminant Validity (HTMT) With 95% Bootstrapped Confidence Intervals (Study 2)**

| **Construct Pair** | **HTMT** | **95% CI LL** | **95% CI UL** |
| --- | --- | --- | --- |
| Professional Growth ↔ Achievement Recognition | 0.281 | 0.206 | 0.387 |
| Teaching Performance ↔ Achievement Recognition | 0.853 | 0.609 | 1.010 |
| Teaching Performance ↔ Professional Growth | 0.774 | 0.550 | 0.963 |
| Time Orientation ↔ Achievement Recognition | 0.453 | 0.387 | 0.515 |
| Time Orientation ↔ Professional Growth | 0.350 | 0.357 | 0.473 |
| Time Orientation ↔ Teaching Performance | 1.014 | 0.803 | 1.224 |

*Note. HTMT < .85 indicates acceptable discriminant validity. Values approaching or exceeding .85 (or >1.00) suggest potential conceptual overlap or limited precision in abbreviated constructs.*

**Table S6. Global Model Fit Indices (PLS-SEM) for Study 2**

| **Index** | **Saturated Model** | **Estimated Model** | **95% CI LL** | **95% CI UL** |
| --- | --- | --- | --- | --- |
| SRMR | 0.063 | 0.063 | 0.065 | 0.067 |
| d_ULS | 6.048 | 6.077 | 6.499 | 6.954 |
| d_G | 1.416 | 1.420 | 1.791 | 1.934 |

*Note.* SRMR values < .08 indicate acceptable model fit. d_ULS and d_G should be interpreted with caution, especially in models including abbreviated or heterogeneous constructs.

**Table S7. Descriptive Statistics and ANOVA Results for the ZTPI-C Future Orientation Subscale by Condition (Study 2)**

| **Condition** | **n** | **M** | **SD** | **95% CI [LL, UL]** |
| --- | --- | --- | --- | --- |
| Control group | 77 | 21.30 | 2.56 | [20.72, 21.88] |
| Short-term Perspective | 73 | 23.73 | 7.42 | [21.99, 25.46] |
| Long-term Perspective | 74 | 27.96 | 5.46 | [26.69, 29.23] |

*Note***.** ANOVA: F(2, 221) = 28.41, p < .001, η² = .21. Tukey post hoc tests: Long-term > Short-term > Control (all ps < .05).

**Table S8. Effect Sizes (Cohen’s d) and 95% Confidence Intervals for Pairwise Comparisons Across Conditions (Study 2)**

| **Achievement Recognition** | **Cohen’s d** | **95% CI** |
| --- | --- | --- |
| Long-term vs. Control | **1.15** | [0.81, 1.50] |
| Short-term vs. Control | **0.54** | [0.21, 0.86] |
| Long-term vs. Short-term | **0.56** | [0.23, 0.89] |
| **Teaching Performance** | **Cohen’s d** | **95% CI** |
| Long-term vs. Control | **1.10** | [0.76, 1.44] |
| Short-term vs. Control | **0.37** | [0.05, 0.70] |
| Long-term vs. Short-term | **0.59** | [0.26, 0.92] |
| **Professional Growth** | **Cohen’s d** | **95% CI** |
| Long-term vs. Control | **1.18** | [0.83, 1.53] |
| Short-term vs. Control | **–0.43** | [–0.76, –0.11] |
| Long-term vs. Short-term | **1.54** | [1.17, 1.91] |

*Note***.** Effect sizes were computed using pooled standard deviations and group sample sizes (n_control = 77, n_short = 73, n_long = 74). Negative values indicate lower scores in the short-term condition relative to the control condition.
